# A histological examination of spinal reconstruction using a frozen bone autograft

**DOI:** 10.1371/journal.pone.0191679

**Published:** 2018-01-25

**Authors:** Kazuya Shinmura, Hideki Murakami, Satoru Demura, Satoshi Kato, Katsuhito Yoshioka, Hiroyuki Hayashi, Kei Inoue, Takashi Ota, Noriaki Yokogawa, Takayoshi Ishii, Takashi Igarashi, Hiroyuki Tsuchiya

**Affiliations:** Department of Orthopaedic Surgery, Graduate School of Medical Science, Kanazawa University, 13–1 Takara-machi, Kanazawa, Japan; Szegedi Tudomanyegyetem, HUNGARY

## Abstract

Our aim was to compare the process of bone formation after reconstruction of the vertebral body using a titanium cage with either a liquid nitrogen-treated (frozen) bone autograft or non-treated fresh bone autograft. Twelve canine beagles underwent anterior reconstruction of the 5^th^ lumbar vertebrae using a titanium cage and bone autograft. Bone formation was compared across four experimental groups: fresh bone autograft groups, with animals sacrificed at either 8 or 16 weeks post-reconstruction, and liquid nitrogen-treated (frozen) bone autograft groups, with animals again sacrificed at either 8 or 16 weeks post-reconstruction. Bone formation was evaluated histologically by calculating the proportion of ‘reaction’ and ‘mature bone’ regions at the ends of the cage, its center, and ventral/dorsal sides. The reaction region contained osteocytes with a nucleus and osteoblasts accumulated on the surface of an osteoid, while a laminar structure was visible for mature bone regions. For fresh bone autografts, the reaction and mature bone regions significantly increased from 8 to 16 weeks post-reconstruction. By comparison, for frozen autografts, the reaction bone region did not significantly increase from 8 to 16 weeks post-reconstruction, while the mature bone region did increase over this time period. The proportion of reaction bone was higher at the ends and dorsal side of the cage at 8 weeks, for both graft types, with greater bone formation at the center of the cage at 16 weeks only for the fresh bone autograft. Therefore, bone formation in the anterior spinal reconstruction site tended to be delayed when using a frozen bone autograft compared to a fresh bone autograft. The bone formation process, however, was similar for both groups, beginning at the ends and dorsal side of the cage adjacent to the surrounding vertebral bone.

## Introduction

With continuing advancement of cancer therapy, acceptable long-term prognosis can be expected even for patients with metastatic spinal tumors [1–4]. As en bloc resection of a tumor-bearing vertebra can be curative, leading to longer-term survival [5,6], achieving bone fusion of the reconstructed vertebral body is essential [7]. Akamaru et al. obtained fusion of the reconstructed vertebra to adjacent vertebral bodies using a titanium cage with a fresh bone autograft harvested from the ilium, with evidence of remodeling at 16 months post-reconstruction in autopsy samples [8]. However, harvesting of bone from the ilium is not an inconsequential procedure, with a risk of post-operative pain at the site of harvest, among other possible comorbidities [9]. To address these issues, Murakami et al. treated the resected tumor-bearing vertebra using liquid nitrogen and subsequently used the crushed treated bone as a substitute to fresh bone autograft in the titanium cage for vertebral body reconstruction. This method does not require bone harvesting from the ilium, with the freezing of the tumor-bearing vertebra providing a systemic anti-cancer immunity [10,11].

Promising results of cryosurgery have been reported for some malignant tumors [12–14], with the tumor cells being completely destroyed by liquid nitrogen, while the remaining tumor antigens released from the necrotic tumor cells activate a tumor-specific immune response [15]. However, osteocytes and osteoblasts, which play an important role in bone fusion, are also destroyed in the cryogenic process and, therefore, it is not clear that bone fusion of the reconstructed vertebral body can be achieved. Although Garusi et al. reported that bone autograft frozen by liquid nitrogen acts in the same way as a normal graft in rat mandible [16], there is a lack of histological studies regarding the process of bone formation using crushed liquid nitrogen-treated bone autografts for large bone defects. Thus, the aim of our study was to compare the process of bone formation after reconstruction of the L5 vertebral body using a titanium cage with either a liquid-nitrogen treated bone autograft or a fresh bone autograft in a canine model.

## Methods and procedures

### Animals

This study was conducted with approval from the Committee of Animal Care and Experimentation at Kanazawa University (Kanazawa, Japan, AP-112189). One-year-old female beagle canines (body weight, 10–12 kg) were purchased from Japan SLC (Shizuoka, Japan) at one year of age and acclimated for a few weeks prior to surgery. The dogs were housed separately in a stainless-steel cage with a resin-coated floor in order to reduce the load on their feet, which conforms to the ILAR guideline for the Care and Use of Laboratory Animals. Animals were provided with access to water ad libitum, as well as provided with 200 g of solid feed (DM-2, Funabashi Farm, Chiba) twice daily. The environment was controlled at a temperature of 23±2°C and humidity of 55±10%, with 12 H: 12 H light-to-darkness cycle. The health of the dogs was monitored on a daily basis by a veterinarian who was on site or on call during this phase of the study.

### Surgical procedure

Resection and reconstruction of the L5 vertebral body was performed under sedation, using dormicum (0.2 mg/kg) and medetomidine (20 μg/kg), injected intramuscularly into the paraspinal muscles. Following sedation, a venous catheter was placed in the cephalic vein to prepare a path for fluid replacement. Propofol (3 mg/kg) was administered intravenous, followed by intubation, using a 6.5-mm cuff inserted through the mouth and connected to a ventilator (R-10, AIKA, Tokyo), providing controlled ventilation with a 50/50% mixture of oxygen/nitrous oxide. General anesthesia was maintained by continuous intravenous administration of propofol (8 mg/kg/h) and pancuronium, appropriately administered intravenously to maintain the animal still. Following anesthesia, ceftriaxone (1 g) was injected intravenously. The animal was then placed in the left lateral decubitus position and resection of the L5 vertebral body was performed, in combination with resection of the L4/5 and L5/L6 intervertebral discs, using a retroperitoneal approach. The resected L5 vertebral body was used as bone graft. A titanium cage (diameter, 12 mm and length, 27 mm; Depuy AcroMed, Raynham, MA) was filled with either a crushed non-treated fresh bone autograft, used as the control, or a crushed frozen bone autograft. For the freezing procedure, the resected vertebral body was soaked in liquid nitrogen (-196°C) for 20 min.

**Fig 1:**
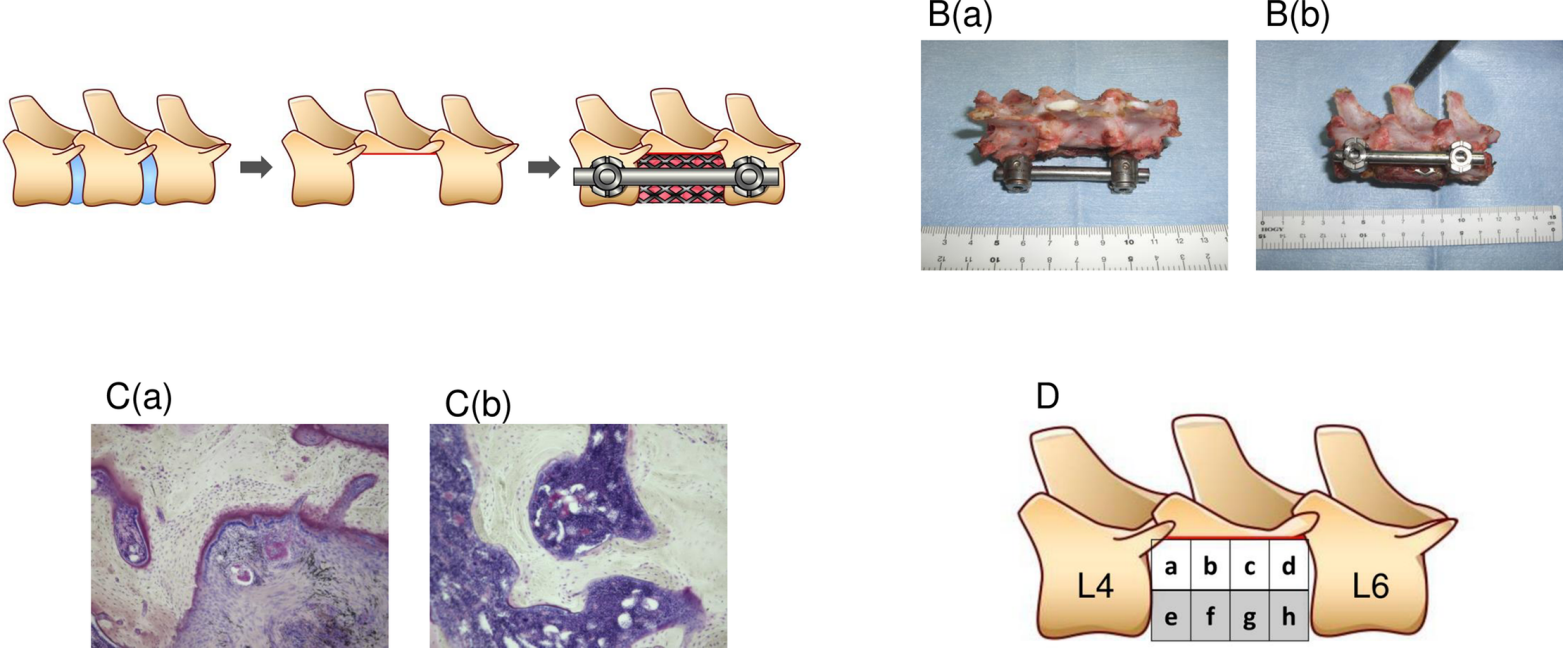
**A.** The L5 vertebral body is resected, along with the L4/5 and L5/L6 intervertebral discs, and a cage, filled with either a crushed non-treated fresh bone autograft or liquid nitrogen-treated bone autograft, is inserted into the site of the resected L5 vertebral body. Screws inserted into the vertebral bodies of L4 and L6 were connected to a titanium rod to fix the cage *in situ* by compression. **B.** Excised specimens showing the dorsal (a) and lateral (b) sides. **C.** Histological samples with Villanueva bone stain, showing (a) the reaction region, with osteocytes having a nucleus, a formed osteoid and an accumulation of osteoblasts on the surface of the osteoid and (b) the mature bone region, with the lamellar structure being visible. **D.** A schematic representation of the specimen, showing division of the area within the cage into 8 sections for analysis.

The filled titanium cage was inserted into the site of the resected L5 vertebral body. Pedicle screws (diameter, 5.5 mm; Depuy AcroMed, Raynham, MA) were inserted into the vertebral body of L4 and L6 and connected to a titanium rod (diameter, 5.5 mm; Depuy AcroMed, Raynham, MA) to fix the titanium cage via compression, by shortening the distance between the screws (Fig 1A). Both the fascia and skin were approximated and sewn using 3–0 silk thread to close the incision. Buprenorphine was administered intramuscularly (0.01 mg/kg) to manage pain every 12 H, beginning at the time of sedation, for up to 3 days post-surgery. After surgery, ventilation was managed until spontaneous breathing resumed, and the intubation tube was removed once consciousness and the respiratory status were judged to be satisfactory. In order to prevent infection, ceftriaxone (0.5 g) was injected intramuscularly in one daily dose up to post-operative day 3.

### Experimental groups

Dogs were randomly divided into the following 4 groups, according to the type of bone graft used for the anterior reconstruction and the time period up to sacrifice, with 3 dogs per groups. 1) fresh bone autograft, sacrificed at 8 weeks post-reconstruction (C-8w group); 2) fresh bone autograft, sacrificed at 16 weeks post-reconstruction (C-16w group); 3) frozen bone autograft, sacrificed at 8 weeks post-reconstruction (F-8w group); and 4) frozen bone autograft, sacrificed at 16 weeks post-reconstruction (F-16w group).

### Specimen preparation

After propofol (3 mg/kg) was intravenously administered to sedate the animal, 20 mL of 1 M potassium chloride was administered intravenously for sacrifice. Immediately after sacrifice, the spinal segment from L4 to L6, including the cage, was removed en bloc (Fig 1B). The excised samples, from which screws and rod were extracted and the soft tissues dissected, were removed and fixed with 10% formalin and 70% ethanol.

### Histological analysis

Fixes samples were soaked in a Villanueva bone stain solution for 7 days and methyl methacrylate-embedded sections (thickness, 30 μmm) were prepared, by grinding, for analysis.

Two different regions were identified for analysis (Fig 1C): a ‘reaction region’, defined as a region that includes osteocytes having a nucleus, a formed osteoid and an accumulation of osteoblasts on the surface of the osteoid; and a ‘mature bone region’, defined as a region which meets the criteria of a reaction region and in which the lamellar structure is visible.

To quantify the amount of newly formed bone, we calculated the proportional distribution of ‘reaction’ and ‘mature bone’ regions within a cage: (area of the reaction region/area of the entire cage) × 100 (%); and (area of the mature bone region/area of the entire cage) × 100 (%). The proportion of each region was compared between the four experimental groups.

To quantify the process of new bone formation within a cage, each cage was divided into 8 sections (Fig 1D) and the proportional distribution of ‘reaction’ and ‘mature bone’ regions calculated within each region as follows: (area of the reaction region in specified sections/area of the entire cage) × 100 (%); and (area of the mature bone region in specified sections/area of the entire cage) × 100 (%). The calculated proportions were then compared between the following sections; 1) the ends of the cage (sections a, d, e, and h), the center of the cage (sections b, c, f, and g); 2) the dorsal (sections a, b, c, and d) and ventral (sections e, f, g, and h) regions. All areas were measured using ImageJ (version 1.46r) software [17–19].

### Statistical analysis

An analysis of variance (ANOVA) was used to evaluate between-group differences in the proportion of reaction and mature bone regions. Differences in these proportions, by area of the cage, were evaluated using a *t*-test. For all analyzes, a *P-*value < 0.05 was considered to be statistically significant.

## Results

### Histological findings

**Fig 2:**
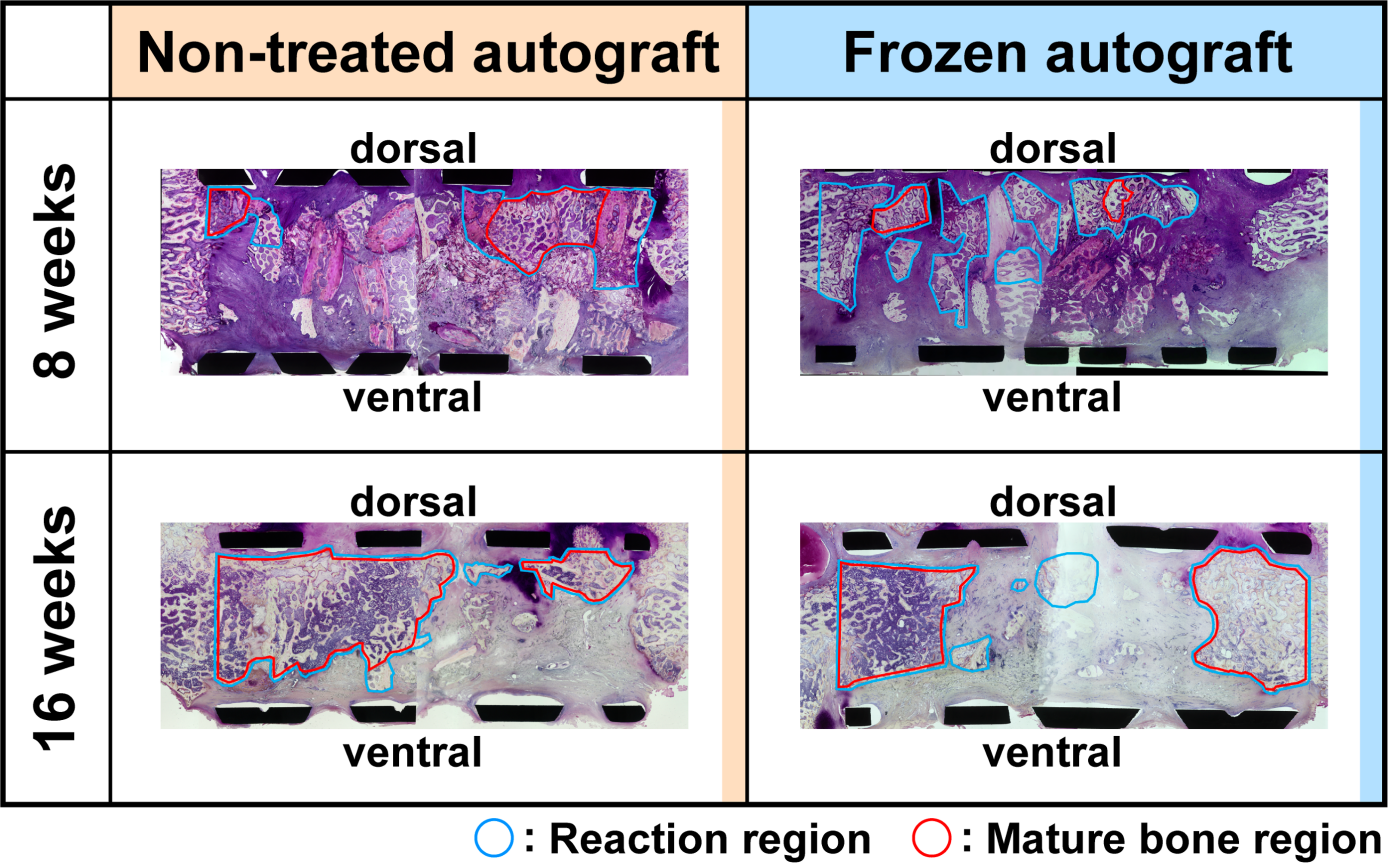
Histological images for each group, showing reaction regions (blue circle) and mature bone regions (red circles). The area of both regions increased from 8 to 16 weeks post-reconstruction in both fresh and frozen bone autograft.

At 8-weeks, reaction and mature bone regions were sparse for both fresh and frozen bone autografts, with both regions expanding at 16 weeks post-reconstruction (Fig 2).

**Fig 3:**
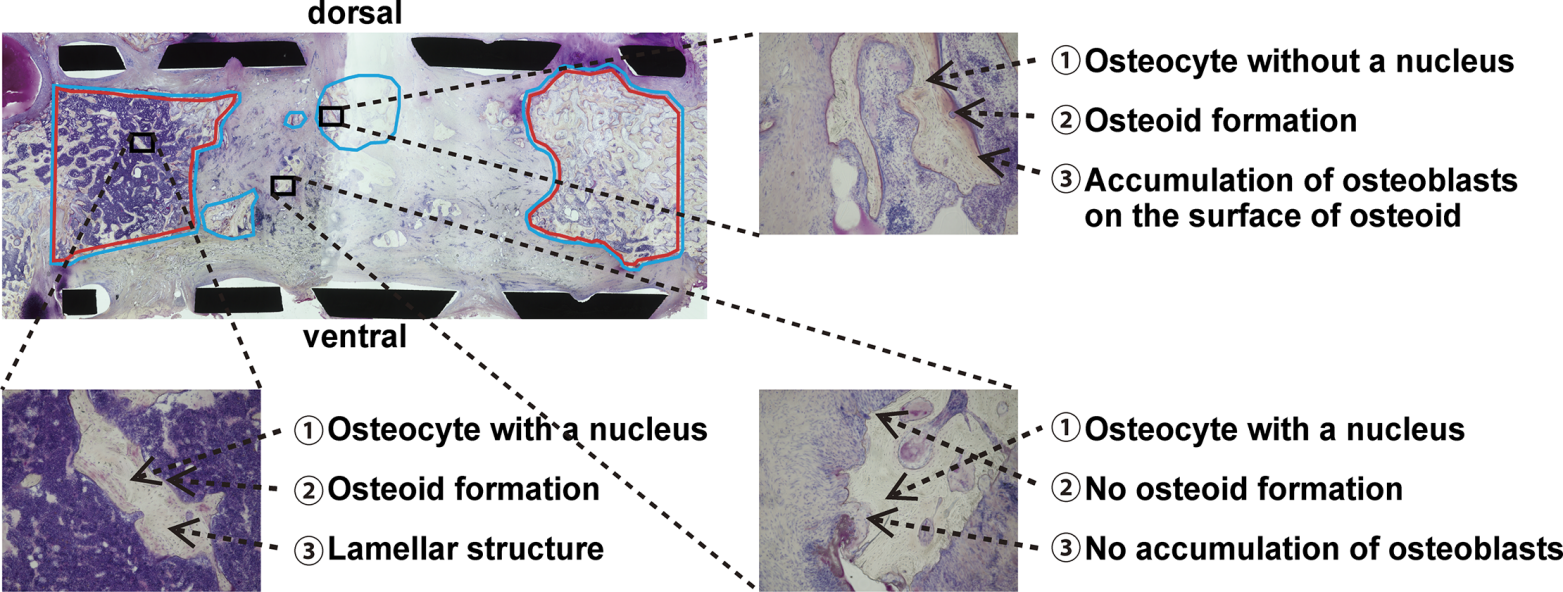
Representative histological images at 16-week post-reconstruction for the liquid nitrogen-treated bone autograft. Mature bone was identified in the ends of the cage, while only reaction regions were visible on the dorsal side in the center of the cage, with no osteocytes, accumulation of osteoblasts or formation of osteoid being visible on the ventral side.

In the group F-16w, mature bone was identified in the ends of the cage, while only reaction regions were visible on the dorsal side in the center of the cage (Fig 3).

### Analysis of newly formed bone

**Fig 4:**
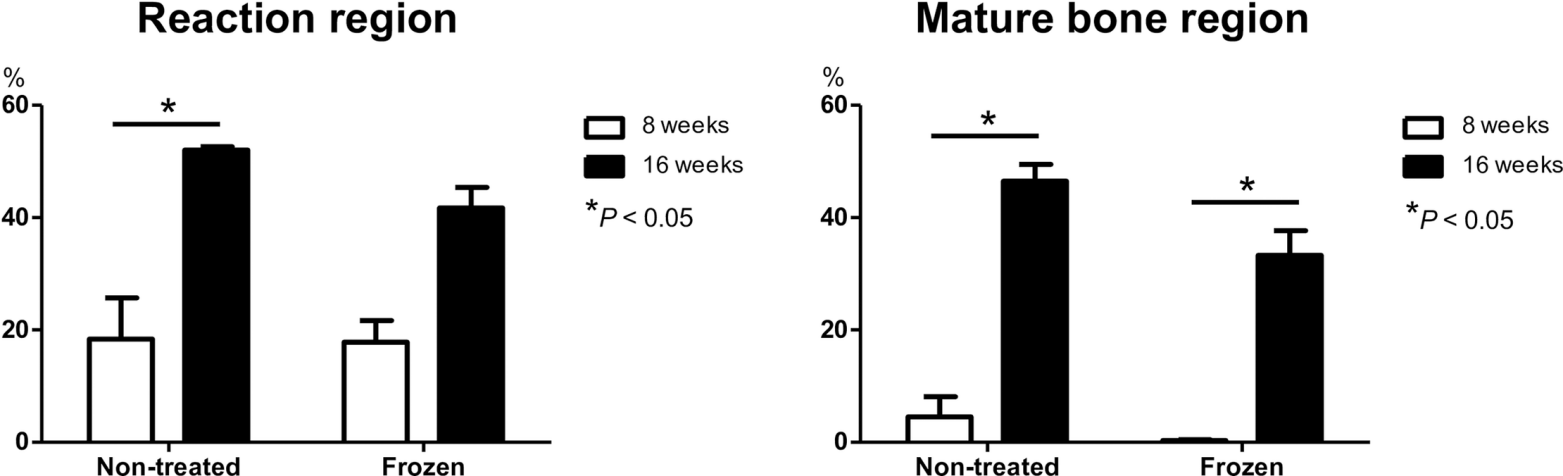
The proportion of the ‘reaction’ and ‘mature bone’ region among the 4 experimental groups are shown, including the mean±standard deviation. Note that while both reaction and mature regions increased from 8 to 16 weeks post-reconstruction for the fresh bone autograft groups, only an increase in mature regions were observed in the frozen bone autograft groups.

The proportion of reaction regions across the 4 experimental groups is reported in Fig 4, and summarized as follows: C-8w group, 18.3%; C-16w group, 52.0%; F-8w group, 17.8%; and F-16w group, 41.7%. The proportion of reaction regions increased from 8 to 16 weeks post-reconstruction for both the fresh and frozen bone autografts, with this increase being significant for the fresh bone autografts. The proportion of mature bone region across the 4 experimental groups was as follows: C-8w group, 4.5%; C-16w group, 46.5%; F-8w group, 0.3%; and F-16w group, 33.3%. The mature bone region significantly increased from 8 to 16 weeks post-reconstruction in the both bone graft types.

**Fig 5:**
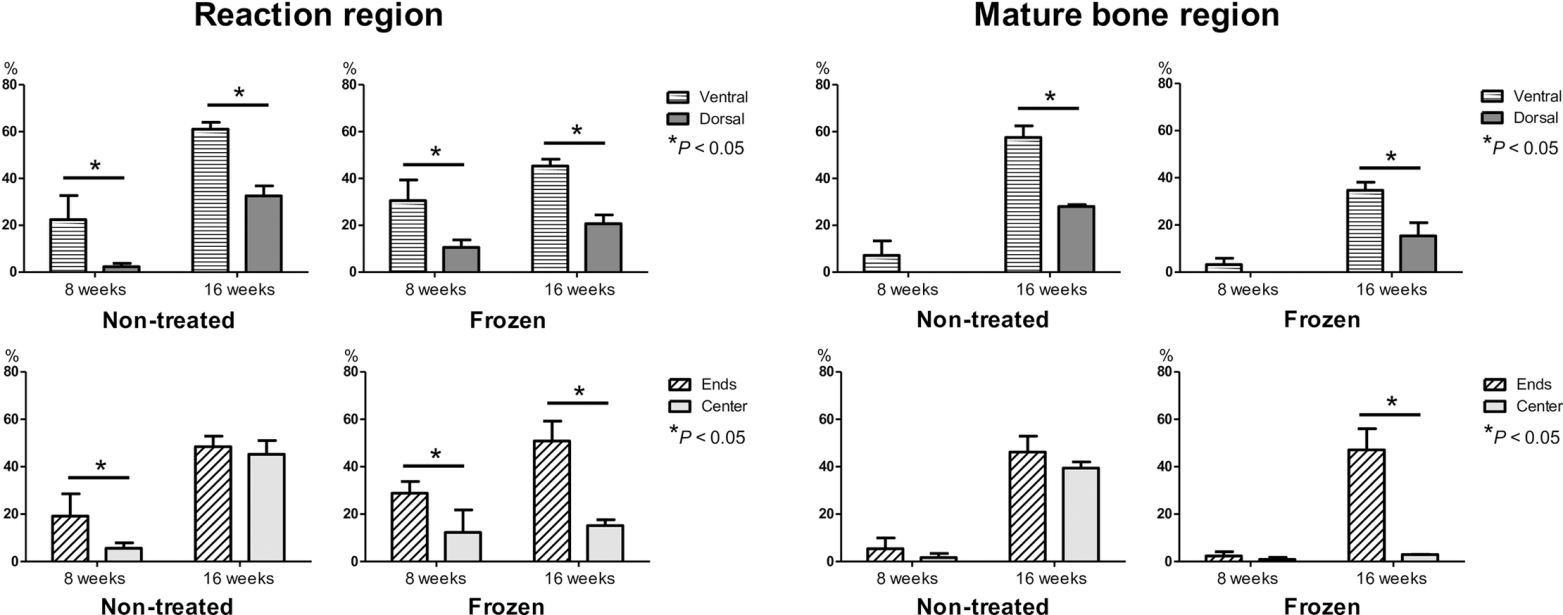
The amount of newly formed bone in each of the 8 sub-sections of the cage is graphed with mean±standard deviation for each group. The proportion of reaction regions is significantly higher in the ends of the cage compared to its center region, as well as on the dorsal region compared to the ventral region in the both 8-week groups. In contrast, the C-16w group has a higher proportion of reaction regions in the center of the cage, while the F-16w group shows little increase in this section, while the proportion of mature regions was similar between the two groups.

The amount of newly formed bone in each of the 8 sub-sections of the cage is shown in Fig 5. The proportion of reaction regions was significantly higher in the ends of the cage compared to its center region, as well as on the dorsal region compared to the ventral region in the both 8-week groups. The C-16w group had an increase proportion of reaction regions in the center of the cage, with the difference in the proportion of reaction region between the center and ends of the cage no longer being significant. In contrast, the proportion of reaction regions in the F-16w group increased in the ends of the cage, with a small increase in the center of the cage. We obtained similar result for the proportion of mature bone regions. Taken together, our analysis showed that bone formation and maturation starts at the ends and dorsal side of a cage, and subsequently extends towards the center and ventral side of the cage in both graft types. In addition, bone formation and maturation in the center of a cage at 16 weeks are more favorable for fresh than frozen bone autografts.

### Additional examination

**Fig 6:**
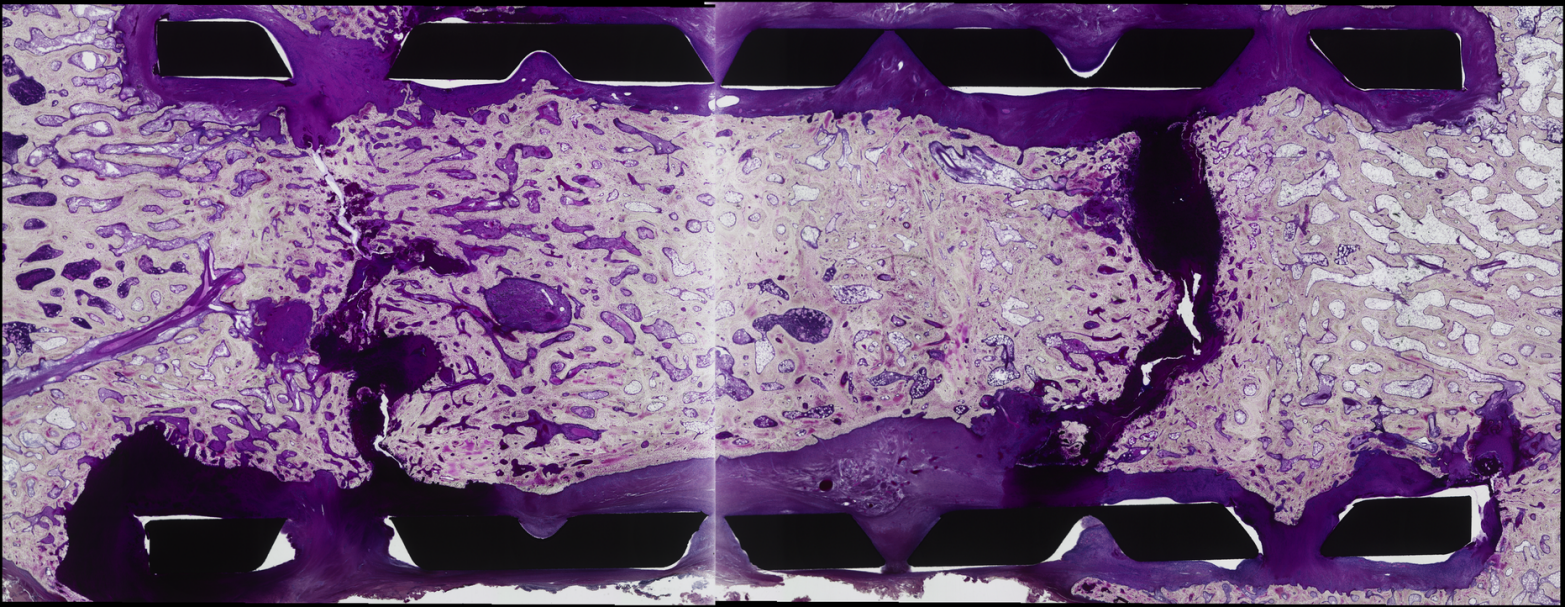
A histological image of the 1-year model of the frozen bone autograft, showing remodeled bone filling the entire cage including center region.

To evaluate if frozen bone autograft undergoes complete remodeling, an additional frozen autograft dog model was sacrificed at a year post-spinal reconstruction. As shown in the Fig 6, the entire region of the cage was filled with remodeled mature bone.

## Discussion

We describe an anterior-spinal reconstruction dog model using a titanium cage with 2 types of autologous crushed bone graft: fresh and liquid nitrogen-treated autograft. In this model, bone formation in the cage tended to be delayed when using a frozen bone autograft compared to a fresh bone autograft, although similar bone formation process was observed in both autografts, which began at the ends and dorsal side of the cage adjacent to the surrounding vertebral bone.

Various graft types have been used for reconstruction, including fresh autologous bone, liquid nitrogen-treated bone, autoclaved bone, and allograft bone grafts. Since bone morphogenetic proteins (BMPs) play an important role in bone formation, grafts in which BMPs are conserved are useful to achieve bone fusion. Takata et al. compared BMP-7 activity in resected human bone from the femoral head treated at -196°C, -70°C, 60°C, or 100°C, reporting better conservation of BMP-7 activity in bone treated at a temperature of -196°C [20]. Similarly, better preservation of BMPs has been reported to be achieved in bone graft treated at low temperature [21,22]; thus, liquid nitrogen-treated bone could be considered to be the most suitable graft to promote bone fusion.

Since Tsuchiya et al. reported excellent outcomes for reconstructive surgery in which malignant tumor-bearing appendicular and pelvic bone treated with liquid nitrogen was used for reconstruction [23], assessment of bone fusion after reconstructive surgery has been described in several studies. Tanzawa et al. reported histological evidence of bone fusion, including active osteoblasts and osteoclasts in the excised region, after reconstructive surgery using a liquid nitrogen-treated bone graft in 5 patients, with favorable bone formation observed [24]. Shimozaki et al. reported favorable rates of bone fusion in reconstructive surgery using malignant tumor-bearing appendicular bones treated with liquid nitrogen using either a pedicle or free freezing procedure [25]. However, strut bones were used for the reconstruction in these studies.

To our knowledge, our study is the first to have evaluated bone formation outcomes after reconstructive surgery of the vertebral body using a crushed liquid nitrogen-treated bone autograft in a titanium cage. We demonstrated that although the amount of new bone formed, indexed by the proportional area of reactive regions, increased from 8 to 16 weeks after reconstruction using a fresh bone autograft, there was no significant increase in new bone formation over this time period for frozen bone autograft. However, the area of mature bone significantly increased from 8 weeks to 16 weeks after surgery even in the frozen autograft group. Therefore, although bone formation is delayed when using a frozen bone autograft, ultimate bone fusion could be expected. In fact, the additional specimen of the one-year model showed complete remodeling, which histologically proved that a frozen bone autograft could undergo complete fusion in a cage.

We also analyzed the process of bone formation in different areas of a cage. Although the fusion process using ground grafted bones in a small cage has previously been reported for a posterior interbody fusion procedures [26–28], bone fusion with the use of a large cage in a spinal corpectomy has not been reported, even for fresh bone autografts. In our study, we demonstrate that bone formation starts at the ends and the dorsal sides of the cage, and subsequently proceeds towards the center and ventral sides. Moreover, a similar process of bone formation was observed in both types of graft, while bone formation in the center of the cage was delayed when using a frozen bone autograft. In a model of posterolateral fusion, Boden et al. demonstrated that blood flow from the decortication region promoted bone fusion by transporting osteogenic cells [29]. Based on our results, we consider that blood flow from the endplates of the vertebral body provided the necessary osteogenic cells for bone fusion, explaining the fast formation and maturation of bone and myeloid tissues adjacent to the endplates, compared to the later bone fusion achieved at the center of a cage. Also, we used a corpectomy model in which the posterior portion of the vertebral body was partially preserved; therefore, higher blood flow would be provided from this portion and lead to the significant earlier bone formation on the dorsal side of the cage compared to its ventral side.

Although our findings support further development of anterior-spinal reconstruction using liquid nitrogen-treated autologous bone graft, there are some notable limitations of our study. The first is the small sample size. Secondly, we evaluated bone formation in the frozen autograft using intact vertebral body instead of tumor-bearing vertebrae. Thus, additional study is required to further assess this procedure.

## Supporting information

S1 ARRIVE checklist (PDF)
